# Associations between first-trimester intrauterine hematoma and twin pregnancy outcomes: a retrospective cohort study

**DOI:** 10.1186/s12884-020-03528-0

**Published:** 2021-01-11

**Authors:** Wanqing Ji, Bo Hou, Weidong Li, Fang Guo, Ping He, Jie Zheng

**Affiliations:** 1 Department of Obstetrics, Guangzhou Women and Children’s Medical Center, Guangzhou Medical University, Guangzhou, 510623 Guangdong Province China; 2 Departments of Neurosurgery, The Third Affiliated Hospital, Sun Yat-sen University, Guangzhou, 510630 Guangdong Province China; 3 Department of Woman and Child Health Information Guangzhou Women and Children’s Medical Center, Guangzhou Medical University, Guangzhou, 510623 China

**Keywords:** Intrauterine hematoma, Twin gestation, First trimester, Miscarriage, Vanishing twin syndrome

## Abstract

**Background:**

In recent years, first-trimester intrauterine hematoma (IUH) has become increasingly common in twin pregnancy. The majority of studies on IUH have excluded twin pregnancies, and others did not differentiate between singleton and twin pregnancies. The impact of IUH on twin pregnancy is unclear. Therefore, the primary objective of our study was to examine associations between first-trimester IUH and pregnancy outcomes in twin pregnancies.

**Methods:**

The data of 1020 twin pregnancies in women who received a routine examination from January 2014 to December 2018 were reviewed. We compared baseline data and pregnancy outcomes between those with and without IUH. Multivariable logistic regression analysis was used to adjust for possible confounding factors.

**Results:**

A total of 209 patients (21.3%) developed IUH in the first trimester. First-trimester IUH was significantly associated with increased odds of miscarriage (adjusted odds ratio 14.27, 95% CI 8.25–24.70) and vanishing twin syndrome (adjusted odds ratio 3.26, 95% CI 1.11–4.61). However, there were no differences in the rates of stillbirth, preeclampsia, preterm labor (< 34 weeks), low birth weight, postpartum hemorrhage or fetal distress between the two groups. Maternal age, previous preterm birth, chorionicity in twins and the gestational week at first ultrasound did not differ between the two groups. The women with IUH had high rates of previous miscarriage (46.73% vs 38.37%, *p* = 0.01), assisted conception (48.56% vs 32.60%, *p* < 0.001) and accompanied vaginal bleeding (67.46% vs 13.43%, *p* < 0.001). According to the logistic regression analyses, these characteristics were not associated with pregnancy loss or vanishing twin syndrome. No IUH characteristics, including volume, largest diameter, or the presence of vaginal bleeding, were associated with pregnancy loss or vanishing twin syndrome before 20 weeks of gestation (*P* > 0.05).

**Conclusion:**

In women with twin pregnancy, the presence of IUH in the first trimester was associated with the loss of one or both fetuses before 20 weeks of gestation. However, previous miscarriage, the conception method, the IUH size and the presence of vaginal bleeding were not independently associated with miscarriage or vanishing twin syndrome.

## Background

Intrauterine hematoma (IUH) is a common finding on ultrasound examinations, with the reported incidence varying widely, from 0.46 to 39.5% [1, 2]. A total of 62.9% of hematomas occur during the first trimester and usually disappear within 3 months after detection [3]. One of most commonly reported adverse outcomes associated with IUH is pregnancy loss [4, 5]. Tuuli et al. reported an increased risk of pregnancy loss in women with subchorionic hematoma (17.6% vs 8.9%) in a meta-analysis published in 2011 [6]. Other studies suggest that IUHs in singleton pregnancies are associated with an increased risk of pregnancy-induced hypertension, preeclampsia, placental abruption and small-for-gestational-age neonates [7, 8]. On the other hand, some researchers have reported that first-trimester subchorionic hematoma was not independently associated with pregnancy loss before 20 weeks of gestation [9] or with adverse pregnancy outcomes after 20 weeks of gestation [10]. However, these results were based on singleton pregnancies.

With the development and use of assisted reproductive techniques, the incidence of twin pregnancy is increasing. It has been reported that assisted reproductive techniques contributed to 16.4% of all multiple-birth infants, and approximately 30.4% of assisted reproductive techniques conceived infants were twins in 2016 [11]. In recent years, we have found that first-trimester IUH in twin pregnancy has become increasingly common. Twin pregnancy is associated with a higher incidence of maternal-fetal complications and more adverse pregnancy outcomes, such as early miscarriage, premature birth, preeclampsia, prenatal bleeding, postpartum bleeding, intrauterine growth restriction and stillbirth, than singleton pregnancy [12]. Pregnancy loss and the disappearance of embryos are the most common adverse outcomes in the first trimester. It has been reported that in women with normal twin pregnancy, approximately 30% of pregnancies will become singleton pregnancy, and 10% will result in the loss of both fetuses [13–15]. The disappearance of gestational sacs or embryos after documented fetal heart activity in multiple pregnancy is known as the vanishing twin phenomenon [16]. The vanishing twin phenomenon is likely associated with a chromosomal abnormality [17]. However, whether IUH leads to pregnancy loss or the vanishing twin phenomenon is unclear. The majority of studies on IUH have excluded twin pregnancies, while others did not differentiate between singleton and twin pregnancies. Therefore, the primary objective of our study was to examine the associations between first-trimester IUH and pregnancy outcomes in twin pregnancy.

## Methods

### Study population

We performed a retrospective analysis of mothers who had two gestational sacs at the first-trimester ultrasound at Guangzhou Women and Children’s Medical Center from January 2014 to December 2018. These women received ultrasound scans at 5 0/7–13 6/7 weeks. Hematoma was diagnosed when crescent-shaped, sonolucent fluid collection behind the fetal membranes or the placenta was observed on a B-ultrasound. The frequency of ultrasound monitoring was based on clinical symptoms and situations. Gestational age was calculated based on the last menstrual period or first-trimester ultrasound scan, per standard guidelines [18]. For the women who underwent assisted reproductive techniques, the gestational week was calculated according to the time of embryo implantation. Twin pregnancy was defined as the presence of two gestational sacs. Viability was confirmed by the presence of fetal cardiac activity on a transvaginal ultrasound at 6 to 7 weeks of gestational age. Chorionicity was identified by the lambda sign and the T-sign on a transvaginal ultrasound scan performed at 7–9 weeks gestation or routine scan at 10–14 weeks [19].

We excluded pregnancies with fetal or placental abnormalities, hematoma found after an operation (multifetal pregnancy reduction, MPR), or elective pregnancy termination. The patients were divided into adverse pregnancy (AP) and normal pregnancy (NP) groups according to the presence or absence of IUH in the first trimester.

### Data collection

We reviewed the electronic medical record for each woman to obtain demographic and clinical information and the ultrasonography findings. Patient demographic data, including maternal age, parity, and previous miscarriage, were collected. Medical record data on the gestational age at first detection of IUH, accompanying symptoms, chorionicity of twins, conception method and pregnancy results were reviewed. The hematoma volume was estimated by measuring the maximum transverse, anteroposterior, and longitudinal diameters and multiplying these values by the constant 0.52, as suggested by Campbel [20]. A correction factor of 0.52 was used to correct for the crescent shape of the hematoma. All measurements were performed with a GE Voluson E8 system (GE Healthcare, Milwaukee, WI, USA) by experienced physicians.

Maternal and neonatal outcomes were recorded as pregnancy outcomes. The outcomes included spontaneous abortion, vanishing twin syndrome (i.e.,the heart of one fetus stopped beating before 14 weeks), preterm delivery at less than 34 weeks of gestation, postpartum hemorrhage, preeclampsia, low birth weight (selective intrauterine growth restriction or twin-to-twin transfusion syndrome were excluded), stillbirth (fetal demise at 20 weeks or more gestational age) and fetal distress. Fetal distress was defined as persistent late decelerations or other heart rate patterns consistent with fetal hypoxia. In addition, low birth weight was defined as < 2500 g.

### Statistical analysis

Quantitative characteristics are described as means ± standard deviations. A t-test was used for comparisons between the AP and NP groups. Qualitative characteristics are described as numbers (percentages), and the chi-square test was used for comparisons between the AP and NP groups. We compared the three groups stratified by IUH size using the Kruskal-Wallis test or chi-square test. The associations between IUH and pregnancy loss were estimated using logistic regression analyses. Initially, unadjusted analyses estimated crude odds ratios and 95% confidence intervals (CIs) (model 1). Multivariable logistic regression analysis was used to adjust for possible confounding factors. We carried out data analyses using SAS version 9.4 (SAS Institute Inc., Cary, NC); p levels were significant at less than 0.05.

## Result

**Fig. 1:**
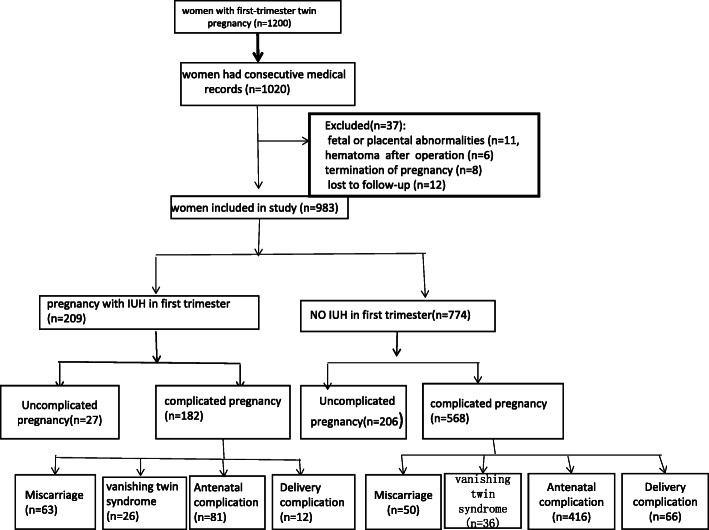
Flowchart showing the study selection of women with twin pregnancy and the incidence of pregnancy outcomes according to the presence or absence of intrauterine hemorrhage. IUH=Intrauterine hemorrhage. Antenatal complications include stillbirth, preterm labor, preeclampsia, and low birth weight. Delivery complications include postpartum hemorrhage and fetal distress

This was a retrospective study. During the study period, 1200 pregnant women had twin pregnancies. A total of 1020 pregnant women had consecutive medical records in our hospital, and their records were reviewed. From these patients, we excluded those with fetal or placental abnormalities (*n* = 11), hematoma found after an operation (multifetal pregnancy reduction, MPR) (*n* = 6), or elective termination of pregnancy (*n* = 8) and those who were lost to follow-up (*n* = 12). Therefore, a total of 983 women were included in the final analysis. A total of 209 (21.3%) patients developed IUH in the first trimester (AP group), and 774 (78.7%) did not develop IUH (NP group) (Fig. 1).

Regarding the baseline characteristics of the women, there were no differences in maternal age, previous preterm birth, chorionicity in twins or the gestational week at the first ultrasound scan between the two groups (Table 1). The NP group had a higher rate of previous term delivery (*p* = 0.01) than the AP group. The women in the AP group had high rates of previous miscarriage (*p* = 0.03), assisted conception (*p* < 0.001) and vaginal bleeding (*p* < 0.001) (Table 1). However, in the logistic regression analyses, the associations were no longer significant (Tables 2 and 3). Previous term delivery, assisted conception and vaginal bleeding may not be major contributions to pregnancy loss or vanishing twin syndrome before 20 weeks.

**Table 1:** Maternal characteristics Based on the Presence or Absence of an Intrauterine Hematoma Before 14 Weeks of Gestation

Characteristics	AP (***N*** = 209) N(%)	NP (***N*** = 774) N(%)	***P*** value
**Maternal age**	30.00 ± 4.31	30.55 ± 4.70	0.127
**Previous term delivery**	52 (26.13)	276 (35.66)	0.01
**Previous Preterm birth**	13 (6.22)	38 (4.90)	0.24
**Previous miscarriage**	93 (46.73)	297 (38.37)	0.03
**Conceived methods**			< 0.001
**Natural conception**	107 (51.44)	521 (67.40)	
**Assisted conception**	101 (48.56)	250 (32.60)	
**Chorionicity of twin**			0.22
**MCDA**	53 (25.36)	236 (31.01)	
**DCDA**	152 (72.73)	512 (67.28)	
**Accompanied bleeding**	141 (67.46)	104 (13.43)	< 0.001
**Gestational age at first scan (wk)**			0.83
**5 0/7–5 6/7**	38 (18.18)	125 (16.15)	
**6 0/7–6 6/7**	40 (19.14)	132 (17.12)	
**7 0/7–7 6/7**	41 (19.62)	148 (19.14)	
**8 0/7–8 6/7**	44 (21.05)	155 (20.02)	
**9 0/7–9 6/7**	19 (9.09)	92 (11.89)	
**10 0/7–10 6/7**	8 (3.82)	33 (4.26)	
**11 0/7–11 6/7**	7 (3.35)	43 (5.50)	
**12 0/7–12 6/7**	6 (2.87)	28 (3.67)	
**13 0/7–13 6/7**	6 (2.87)	17 (2.25)	

*AP* Adverse pregnancy, *NP* Normal group, *MCDA* Monochorionic diamniotic, *DCDA* Dichorionic diamniotic. Values are the mean and standard deviation or number (percentage) unless otherwise indicated. *P* values were determined with the chi-square test for categorical variables or Student’s t test for continuous variables. *P* < 0.05 indicates statistical significance

**Table 2:** Adjusted Risk of miscarriage Before 20 Weeks of Gestation

Risk factors	Rate of Pregnancy Loss at Less Than 20 wk	Unadjusted OR (95% CI)	Adjusted OR^**a**^ (95% CI)
**Subchorionic hematoma**		6.25 (4.14–9.43)	14.27 (8.25–24.70)
**Yes**	30.14		
**No**	6.46		
**Previous term delivery**		0.88 (0.57–1.35)	0.83 (0.45–1.51)
**YES**	10.37		
**NO**	11.63		
**Previous miscarriage**		1.06 (0.71–1.59)	0.62 (0.36–1.09)
**YES**	11.54		
**NO**	10.98		
**Conceived methods**		0.59 (0.38–0.92)	0.52 (0.26–1.04)
**Natural conception**	13.22		
**Assisted conception**	8.24		
**Accompanied bleeding**		2.12 (1.35–3.31)	1.06 (0.60–1.85)
**YES**	13.29		
**NO**	6.76		

Data are n/N (%) unless otherwise specified

^a^Final regression model included subchorionic hematoma, previous term delivery, previous miscarriage, conceived methods, and accompanied bleeding

**Table 3:** Adjusted Risk of vanishing twin syndrome

Risk factors	Rate of vanishing twin syndrome at Less Than 20 wk	Unadjusted OR (95% CI)	Adjusted OR^**a**^ (95% CI)
**Subchorionic hematoma**		2.23 (1.10–3.52)	3.26 (1.11–4.61)
**Yes**	12.44		
**No**	4.64		
**Previous term delivery**		0.84 (0.53–1.32)	0.75 (0.46–1.21)
**YES**	10.39		
**NO**	8.84		
**Previous miscarriage**		0.59 (0.37–0.93)	0.63 (0.39–1.11)
**YES**	7.18		
**NO**	11.66		
**Conceived methods**		0.53 (0.33–0.97)	0.57 (0.35–1.08)
**Natural conception**	11.62		
**Assisted conception**	6.53		
**Accompanied bleeding**		0.61 (0.38–0.97)	0.84 (0.52–1.38)
**YES**	7.51		
**NO**	11.82		

Data are n/N (%) unless otherwise specified

^a^ Final regression model included subchorionic hematoma, previous term delivery, previous miscarriage, conceived methods, and accompanied bleeding

We compared the pregnancy outcomes between the two groups. In the AP group, 63 patients (30.1%) had miscarriages, and twenty-six patients (12.4%) had vanishing twin syndrome. In the NP group, there were 50 cases (6.5%) of miscarriage and 36 cases (4.7%) of vanishing twin syndrome. There were significant differences between the two groups (*p* < 0.001) (Table 4). Similar to the NP group, the AP group did not have increased odds of stillbirth, preeclampsia, preterm labor (< 34 weeks), low birth weight, postpartum hemorrhage or fetal distress (Table 4). According to the logistic regression analyses, first-trimester IUH was associated with an increased risk of miscarriage (adjusted odds ratio 14.27, 95% CI 8.25–24.70) and vanishing twin syndrome (adjusted odds ratio 3.26, 95% CI 1.11–4.61) (Tables 2 and 3).

**Table 4:** Pregnancy outcomes

Outcome	AP group N(%)	NP group N(%)	***P*** value
**Spontaneous miscarriage**	63 (30.14)	50 (6.46)	< 0.001
**Vanishing twin syndrome**	26 (12.44)	36 (4.65)	< 0.001
**Still birth**	2 (0.96)	4 (0.52)	0.61
**Preterm labor(< 34 week)**	35 (16.75)	162 (20.93)	0.26
**Fetal distress**	3 (1.43)	8 (1.03)	0.72
**Preeclampsia**	8 (3.82)	46 (5.94)	0.33
**Postpartum hemorrhage**	9 (4.31)	58 (7.49)	0.10
**Low birth weight (one of twin)**	20 (9.57)	114 (14.75)	0.35
**Low birth weight (both of twin)**	16 (7.78)	90 (11.63)	0.56

*AP* Adverse pregnancy group, *NP* Normal pregnancy group. Values are the mean and standard deviation or number (percentage) unless otherwise indicated. P values were determined with the chi-square test for categorical variables or Student’s t test for continuous variables. *P* < 0.05 indicates statistical significance

We performed a subanalysis of the 209 women with IUH. We compared the IUH features of women who did and did not ultimately experience pregnancy loss (one or two embryos) at less than 20 weeks of gestation (Table 5). We found no associations between IUH volume, IUH diameter or vaginal bleeding and pregnancy loss (one or two embryos) before 20 weeks of gestation.

**Table 5:** Intrauterine hematoma features in women who did and did not have pregnancy loss before 20 weeks of gestation

Intrauterine hematomas feature	Pregnancy Loss at Less Than 20 wk (***n*** = 34)	No Pregnancy Loss at Less Than 20 wk (***n*** = 75)	Vanishing twin syndrome at less than 20wk (***n*** = 15)	***P*** value
Volume (cm3)*	**1.80 (0.77–6.05)**	**1.65 (0.64–3.56)**	**1.61 (0.42–3.47)**	**0.514**^**※**^
**Largest diameter (cm)**	**2.3 (1.7–3.3)**	**2.25 (1.65–2.9)**	**1.95 (1.25–3.1)**	**0.524**^**※**^
**Vaginal bleeding**	**34 (61.82)**	**75 (66.96)**	**15 (62.5)**	**0.499**^**#**^

Data are the median (IQR) or n/N (%) unless otherwise specified

*Measured as length x width x height × 0.52

※Kruskal-Wallis test

#Chi-square test

*P* < 0.05 indicates statistical significance

## Discussion

In this retrospective cohort study, the incidence of hematoma in twin pregnancies reached 21.3%, which is similar to the incidence of hematoma in singleton pregnancies [1, 2]. The main effect of IUH on early pregnancy is pregnancy loss. We found that the presence of first-trimester IUH in twin pregnancy is associated with pregnancy loss or vanishing twin syndrome before 20 weeks of gestation. Maternal age, preterm birth history and chorionicity in twins were not risk factors for IUH. Our data showed that women with twin pregnancies who had a history of miscarriage history, assisted conception, or vaginal bleeding were more likely to have IUHs than their counterparts. However, these factors were not major contributors to pregnancy loss or vanishing twin syndrome before 20 weeks. Our study results are consistent with those of previous research. It has been reported that in vitro fertilization and bleeding were not associated with an increased risk of spontaneous abortion [20–22]. Although recurrent miscarriage is associated with an increased risk of placental previa [23], preterm premature rupture of membranes (PPROM) and preterm delivery [24], we did not find data suggesting that the same risks are associated with IUH.

The clinical impacts of hematoma on first-trimester pregnancy outcomes remain controversial. Many studies have specifically examined the relationship between first-trimester IUH and pregnancy outcomes in singleton pregnancies. A large systematic review reported that IUH was correlated with a twofold increase in spontaneous abortion in singleton pregnancies [6]. However, other studies have found either a lower risk of pregnancy loss [25] or no association between IUH and pregnancy loss [26]. In our study, we found that the fetal loss rate in pregnant women with early IUH was significantly higher than that in women without early IUH; the miscarriage rate was 14.27 times higher, and the vanishing twin syndrome rate was 3.26 times higher than those in women without early IUH. However, first-trimester IUH was not significantly associated with an increased risk of stillbirth, preeclampsia, preterm labor, low birth weight, postpartum hemorrhage or fetal distress in twin gestation. Therefore, timing may be a predictor of pregnancy loss associated with IUH.

In contrast with the pregnancy loss risk in normal early twin pregnancy, IUH is more likely to cause complete pregnancy loss. We found that the risk of total pregnancy loss was notably higher than risk of the disappearance of one twin if the woman had IUH (30.1% vs 12.4%). The exact reason is not clear. It is possible that the effect of IUH on early gestation is “all or nothing”. It has been reported that the vanishing twin phenomenon was associated with preterm delivery, very preterm delivery, small-for-gestational-age neonates and low-birth weight infants [27]. However, we did not explore the differences in the outcomes of pregnancies with a vanishing twin caused by first-trimester IUH, which can be further analyzed in the future.

There are many reports on the relationship between the size of the IUH and pregnancy outcomes, [2, 28, 29]; the effect of IUH size on the rate of pregnancy loss varies across studies [28]. This may be due to the irregular shape of uterine hematomas, which makes measurement difficult. Second, different measurement methods were used, resulting in different conclusions. Howard T et al. compared four methods of measurement and found that subjective hematoma size based on the fraction of the gestational sac size correlated best with first-trimester pregnancy outcomes [28]. In our study, we compiled and analyzed the basic characteristics of the hematomas and found that IUH size was not associated with pregnancy loss or vanishing twin syndrome. Since the subjective evaluation method was difficult to perform in twin pregnancies, three orthogonal hematoma measurements were performed, and the conclusion was the same as that of Mackenzie N et al. [9].

The strength of this study is that it is the first to evaluate the effect of IUH on pregnancy outcomes in patients with twin gestations. However, there were some limitations. First, our study had a retrospective design. The population was collected from a single obstetrics practice, so the data may be subject to regional limitations. Another limitation of this study is that the sizes of the hematomas may have changed after the ultrasound examinations. In addition, persistent IUH may have an impact on pregnancy outcomes, but the duration of IUH was not specifically evaluated in this study.

## Conclusion

In women with twin pregnancy, the presence of IUH in the first trimester is associated with the loss of one or both fetuses before 20 weeks of gestation. In addition, previous miscarriage, the conception method, the IUH size and the presence of vaginal bleeding were not independently associated with miscarriage or vanishing twin syndrome.

## Data Availability

The detailed data sets used and analyzed during the study are available from the corresponding author on reasonable request.

## Abbreviations

IUH: Intrauterine hemorrhage
AP: Adverse pregnancy
NP: Normal pregnancy
MPR: Multifetal pregnancy reduction,
PPROM: Preterm premature rupture of membranes
